# Successful Management of a Tooth With an Endodontic-Periodontal Lesion Using a Bone Graft

**DOI:** 10.7759/cureus.58828

**Published:** 2024-04-23

**Authors:** Manoj Chandak, Payal Chaudhari, Namrata Jidewar, Swayangprabha Sarangi, Anuja Ikhar, Abhilasha Dass, Tejas Suryawanshi

**Affiliations:** 1 Department of Conservative Dentistry and Endodontics, Sharad Pawar Dental College and Hospital, Datta Meghe Institute of Higher Education and Research, Wardha, IND

**Keywords:** reparative, periodontal surgery, surgical endodontics, bone graft materials, endo perio lesions

## Abstract

As periodontal and endodontic tissues have a close association, they come into close touch and have a lot of possible places for communication. In a clinical setting, this correlation promotes infection spread and results in the typical endo-perio lesion appearance. Because the two tissues are in close touch with one another, managing such lesions can be difficult. The success of treatment depends on a thorough examination and careful planning, with the sole focus on repair and regeneration. In these situations, bone graft materials with such characteristics have demonstrated encouraging outcomes. The treatment outcome along with a follow-up for a case of an endo-perio lesion with furcation involvement is shown in the accompanying case report. In treating such instances, a multidisciplinary approach is necessary, emphasizing regeneration.

## Introduction

It has always been unclear, questioned, and contentious how endodontic and periodontal diseases are related to one another. It can be challenging to differentiate between a periodontal issue and an endodontic one [1]. The affected teeth may encounter pain related to the pulp, the periodontium, or both simultaneously. The kind of discomfort and typical symptoms is usually one of the first guides to the problem's cause [2]. A thorough clinical examination and radiographic evaluation can assist in arriving at the correct diagnosis. Pathologies involving the pulp may occasionally have an effect on the periodontium, and vice versa. Diagnosis and treatment planning may become more complex if pulpal issues and inflammatory periodontal disease coexist [3]. The pathogenesis for an endo-perio lesion can be simple or very complex and can take many different forms. Even though it can often be challenging to distinguish between problems of either endodontic or periodontal origin, doing so is crucial to getting the right treatment [4].

## Case presentation

Case history and clinical examination

**Figure 1:**
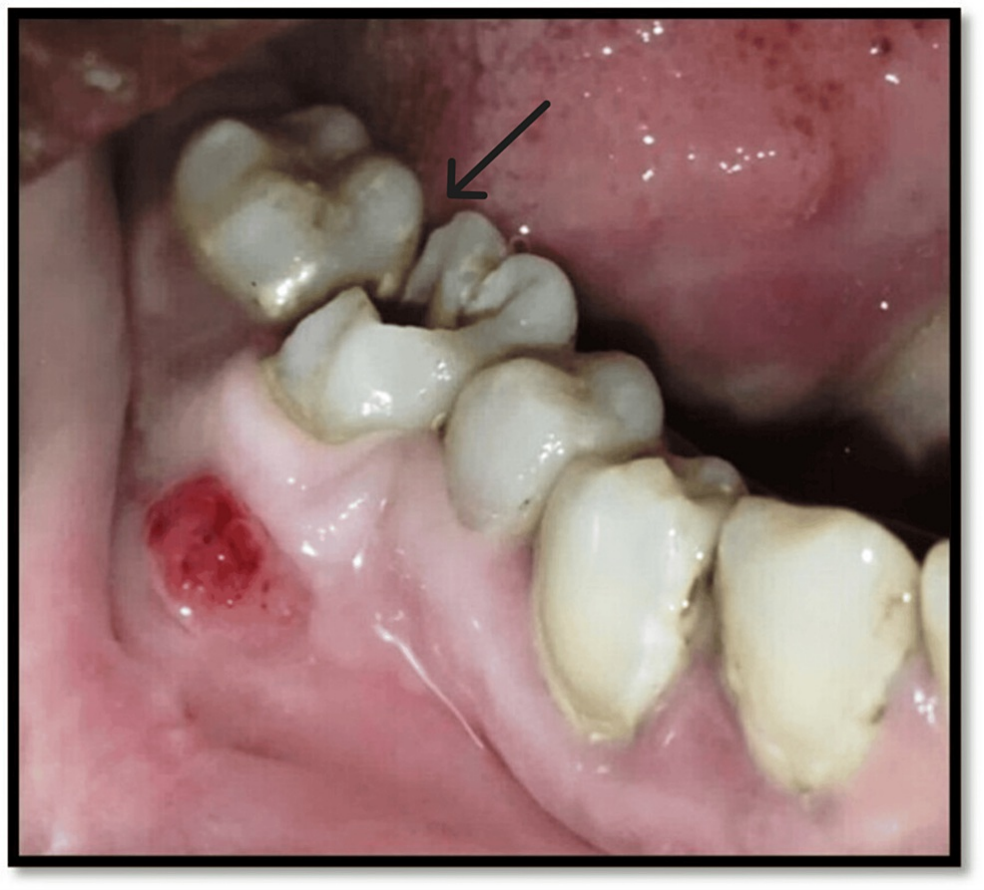
Preoperative intraoral clinical photograph with tooth no. 46 Black arrow highlights the carious lesion

A 41-year-old female patient reported a complaint of pain in the right lower back region of her jaw for one month. Past medical history and past dental history were not significant. Upon intraoral clinical examination, there were deep occlusal caries associated with tooth no. 46 associated with intraoral sinus opening (Figure 1), which showed tenderness on both horizontal and vertical percussion tests associated with sinus. Distoproximal caries were seen with tooth no. 45 and were negative to both vertical and horizontal percussion. Periodontal examination revealed a periodontal pocket of 7 mm deep buccally and 11 mm deep lingually with tooth no. 46.

Investigations

**Figure 2:**
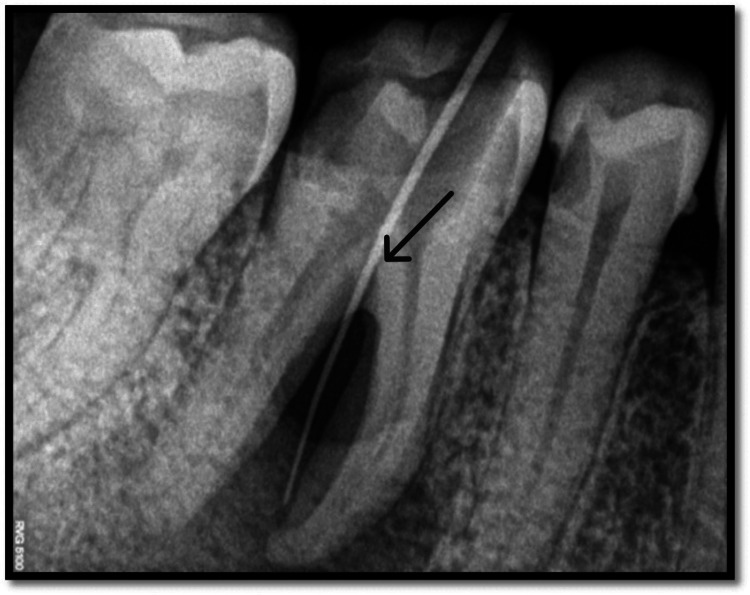
Sinus tract tracing done with tooth no. 46 Black arrow highlights the sinus tracing

**Figure 3:**
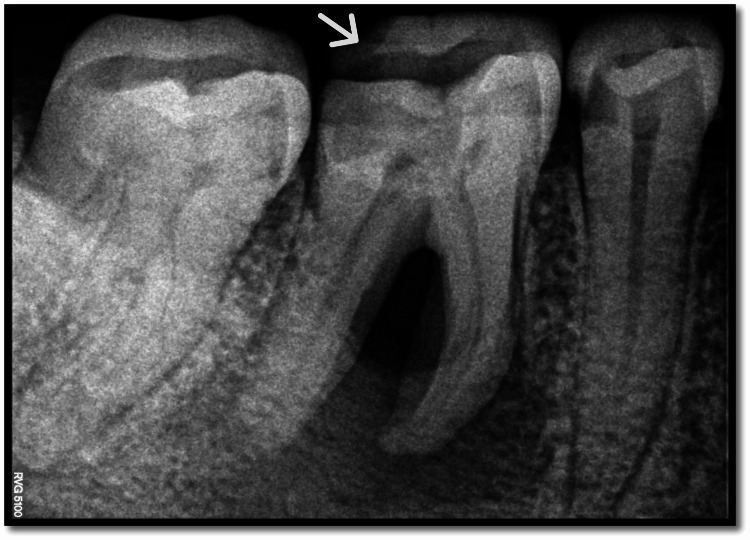
Preoperative radiograph White arrow highlights the tooth involved

Radiographic analysis showed radiolucency involving the pulp, dentin, and enamel. Radiolucency also involved the furcation area which was well defined and rounded. Sinus tract tracing was done with a no. 25 gutta-percha cone (Figures 2-3).

Neural Sensibility Test

The tooth showed no response with both hot and cold test which was further confirmed using electric pulp testing. Electric pulp testing was done in adjunct to thermal testing to check for neural sensibility, which gave confirmation that the tooth was nonvital.

**Figure 4:**
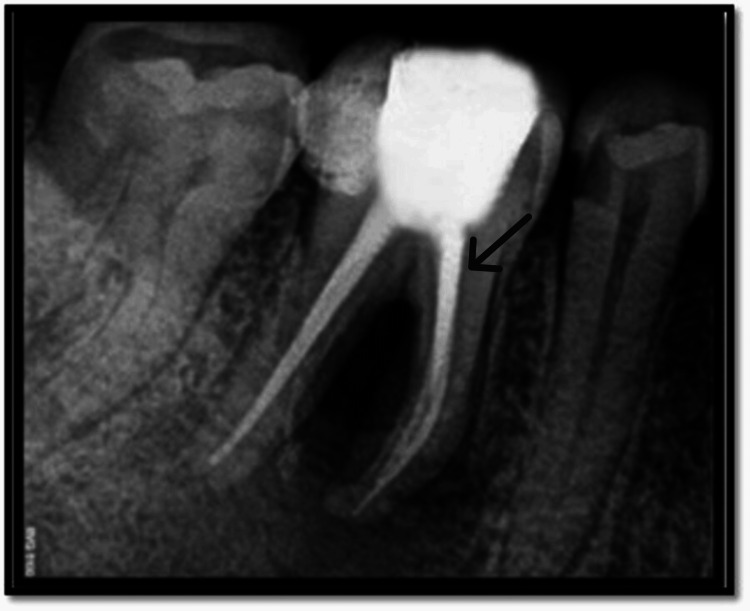
Obturation and post endodontic restoration done with tooth no. 46 Black arrow shows obturation done with tooth no. 46

By combining all the data and aspects right from the history, clinical, and radiographic investigations, a diagnosis of an endo-perio lesion of the primary endodontic origin with secondary involvement of the periodontium was made. Given that this tooth serves a strategic purpose in occlusion and is therefore important strategically, there are only two alternatives left if it is extracted: fixed partial denture (FPD) and implant, but saving a natural tooth has the better prognosis. So a multidisciplinary and interdisciplinary treatment strategy was planned to salvage the tooth. Endodontic therapy was done (Figure 4) prior to periodontal regenerative surgery for the treatment of furcation defect.

**Figure 5:**
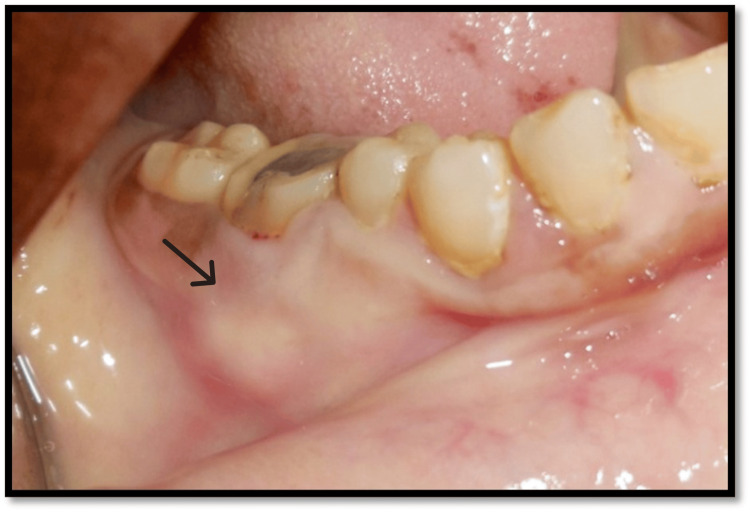
Intraoral photograph of the operative site for bone graft with tooth no. 46 Black arrow highlights the intraoral operative site

**Figure 6:**
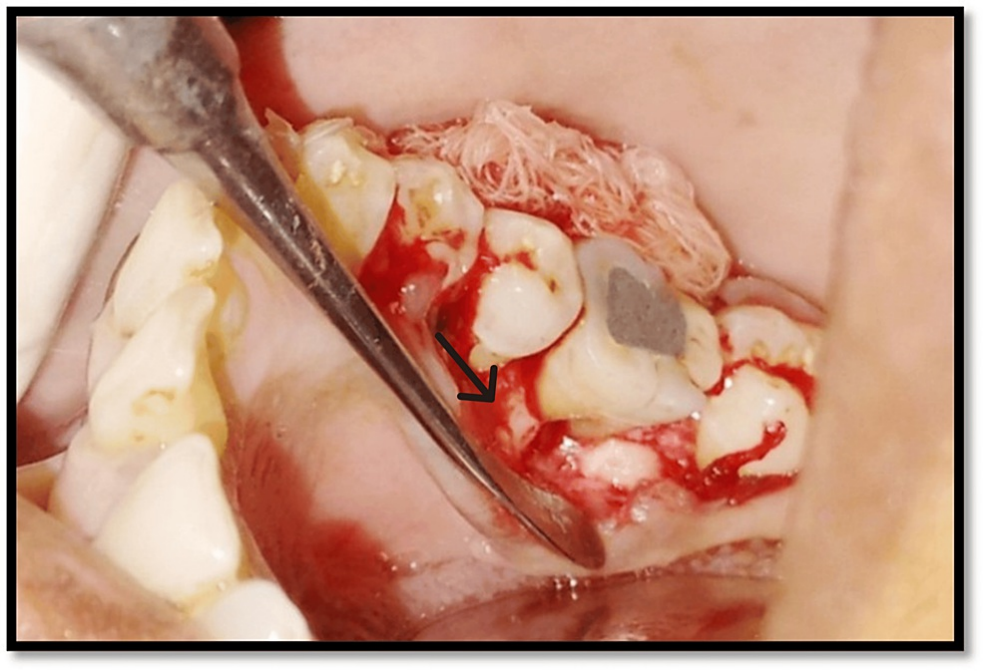
Full-thickness mucoperiosteal flap raised with tooth no. 46 Black arrow highlights the flap raised

The procedure was scheduled once asepsis and sterilization were completed. The preoperative site was evaluated for signs of healing (Figure 5), and after obtaining informed consent from the patient, local anesthesia was achieved using xylocaine and adrenaline at a ratio of 1:80,000 to the surgical site. A “full-thickness mucoperiosteal flap” with the first crevicular incision was elevated from the buccal and lingual side. After some thought, the defect region was thoroughly degranulated and debrided using Gracey's curettes # 13 and 14 (Figure 6).

**Figure 7:**
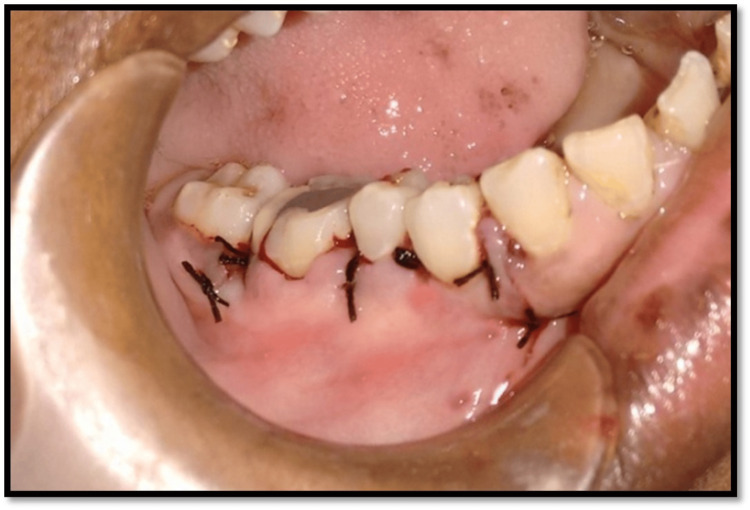
Sutures placed on the surgical site

The bone graft material (comprises sterile hydroxyapatite and tricalcium phosphate granules) was placed in the area with proper bone graft carrier instrument and placed in an incremental manner with condensation following root conditioning of the area using citric acid solution and adequate isolation of the area with proper hemostasis. Sutures in continuous interrupted manner were used to fix the flap (Figure 7), and a periodontal dressing was applied.

**Figure 8:**
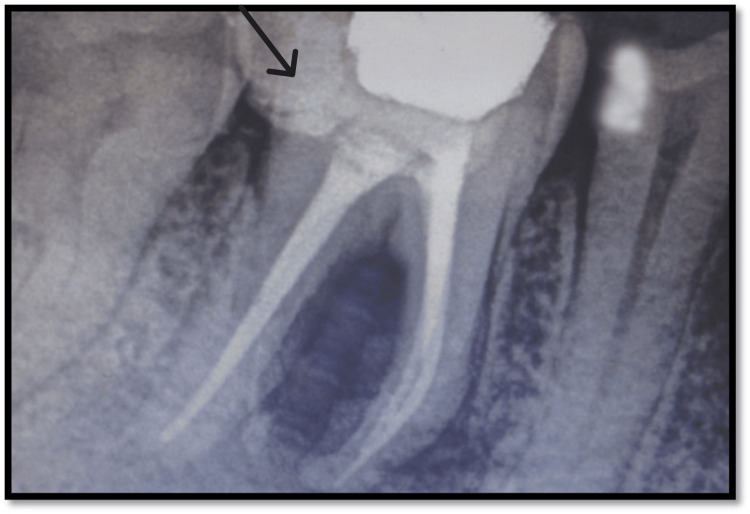
Immediate postoperative radiograph Black arrow highlights the immediate postoperative radiograph of the involved tooth

An immediate postoperative radiograph was taken (Figure 8).

**Figure 9:**
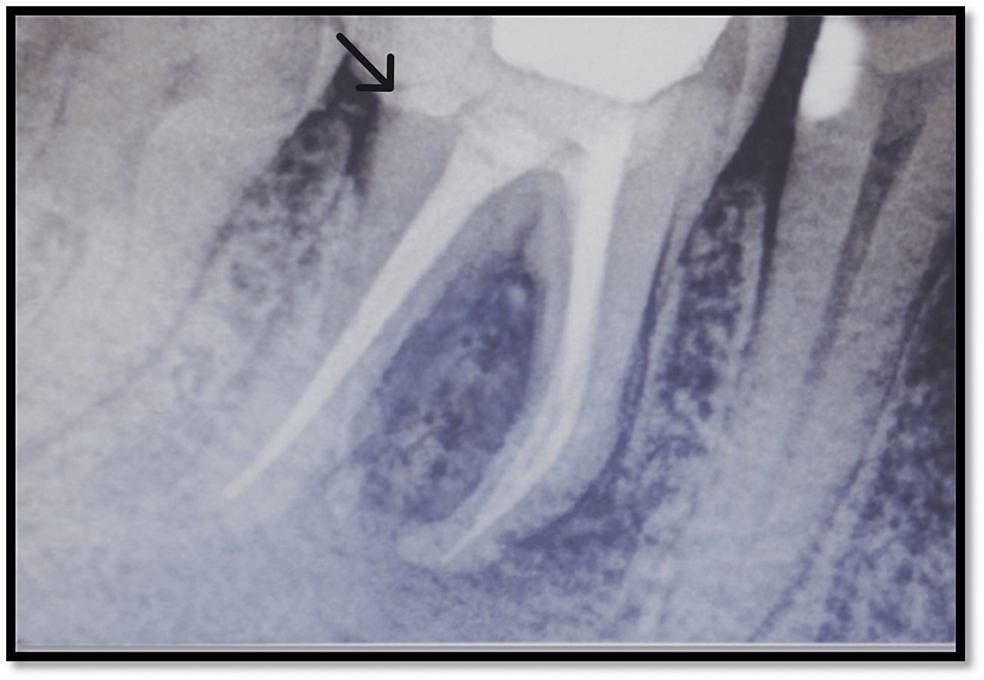
Postoperative radiograph after a six-month follow-up with tooth no. 46 Black arrow highlights the tooth involved after a six-month follow-up

The patient was recalled after 24 hours to evaluate the surgical site, and after seven days, the dressing was removed. Furthermore, the follow-up was continued after one month, three months, and six months, respectively (Figure 9).

## Discussion

The periodontium and the pulp are related on an intimate level, that is “embryonic,” “anatomic,” and “functional” levels. Simring and Golberg gave the first account of their union [5]. The treatment of endo-perio lesions is one of the most typical difficulties in modern therapeutic practice. When pulpal issues and inflammatory periodontal diseases coexist, it might be more difficult to diagnose and establish a course of action for treatment [5,6]. Patients with advanced periodontitis, tooth loss, and pulpal illnesses should be especially aware of this. Since these diseases have mostly been researched as distinct entities, diagnosing them can be difficult because they might mirror the clinical features of other diseases. In order to recognize and precisely determine the contribution of individual lesions to the chief complaint of patient and discomfort, to select the sequence that yields the best outcomes, a full history and meticulous clinical and radiographic evaluation are necessary [7]. When properly carried out, endodontic therapy is extremely predictable and has a high success rate. As long as an endodontic lesion is present, periodontal lesions cannot be treated effectively, and periodontal pockets cannot be removed [8]. No matter how reliable endodontic therapy is, the prognosis for the tooth may be poor if periodontitis has largely destroyed the bone support. Regeneration, root excision [9], and hemisection are indicated in relation to the tactical management of teeth having more than one root. In this instance, endodontic therapy was carried out initially, followed by periodontal therapy and bone grafting for periodontal regeneration.

The pulp vitality test that revealed the tooth's nonvital nature in this report was a crucial discovery that suggested the primary endodontic involvement [10]. An acceptable root canal therapy will typically result in the healing of the endodontic component in the case of mixed endo-perio lesions, and the prognosis will ultimately be dependent upon the effectiveness of the periodontal tissue repair and the regeneration initiated by any of the therapeutic processes. The periodontal lesion in this case did, to a certain extent, recede after endodontic treatment, but not totally, and there was no noteworthy change in the clinical measures. This demonstrated a primary endodontic component and a secondary periodontal involvement.

Calcium hydroxide can be utilized as an intracanal medication when the etiology is only endodontic. It regresses resorption and promotes healing due to its antibacterial, anti-inflammatory, and proteolytic effects [6,10]. It is also helpful in the pathology of endodontic origin with considerable involvement of the periapical area along with pseudo pockets formed due to its temporary obturating-like activity, which avoids the periodontal tissue contamination of the canals after instrumentation via patent lines of communication. The pseudo pocket will typically disappear after following this regimen for a few weeks. However, after endodontic treatment, lesions that are not real mixed lesions would show little to no improvement from the periodontal standpoint, leaving a very poor and frequently hopeless prognosis [9]. However, successful periodontal treatment of such lesions has become achievable with the development of novel regeneration materials [11]. Hydroxyapatite and beta-tricalcium phosphate bone graft materials were chosen since there was a Grade III furcation defect with a deep periodontal pocket. These materials are biocompatible and osteoconductive and provide a surface and chemical environment that promote the production of a new bone [12]. These materials have a low fracture resistance and are fragile. Various preparation techniques result in a material that is either compact or porous with interconnecting macropores that is structurally and spatially equivalent to a cancellous bone. Under normal physiological circumstances, a commercially available hydroxyapatite is resorbed very slowly, whereas beta-tricalcium phosphate is typically resorbed six weeks after implantation. Biodegradable ceramics have the capacity to dissolve, degrade, and unhinderedly permit fresh bone growth and remodeling necessary to achieve optimal mechanical strength [13]. Osteoblasts use hydroxyapatite crystals and beta-tricalcium phosphate as a scaffold on which to build bone while maintaining space for regenerative processes [14]. Following periodontal surgery, epithelial attachment required one month to establish, and full bone growth will take place at the end of the sixth month. The maintenance stage begins even before surgery, and it is the dentist's obligation to stress the value of periodontal therapy. The patient's commitment to maintaining good dental hygiene is crucial to the treatment's success. For a better treatment result, the patient should receive oral hygiene instruction [15,16].

## Conclusions

Teeth with combined endo-perio issues can be challenging to diagnose, necessitating a thorough medical history and the use of a variety of diagnostic tools. For lesions with several etiology, endodontic and periodontal therapy are also necessary. Additionally, regeneration methods, root excision, and hemisections provide different strategies, improving the clinician's capacity to handle these challenging clinical issues.
